# Characterization and Preparation of Nano-porous Carbon Derived from Hemp Stems as Anode for Lithium-Ion Batteries

**DOI:** 10.1186/s11671-019-3161-1

**Published:** 2019-11-07

**Authors:** Zhongxiang Guan, Zhiping Guan, Zhigang Li, Junhui Liu, Kaifeng Yu

**Affiliations:** 1 Key Laboratory of Automotive Materials Ministry of Education, College of Material Science and Technology, Jilin University, Changchun, 130022 China; 2 Institute of Superplasticity and Plasticity of Jilin University, Changchun, 130022 People’s Republic of China; 3 The State Key Laboratory of Inorganic Synthesis and Preparative Chemistry, Jilin University, Changchun, 130022 China

**Keywords:** Hemp stems, Activated carbon, Porous structure, Lithium-ion batteries, High specific capacity

## Abstract

As a biomass waste, hemp stems have the advantages of low cost and abundance, and it is regarded as a promising anode material with a high specific capacity. In this paper, activated carbon derived from hemp stems is prepared by low-temperature carbonization and high-temperature activation. The results of characterizations show the activated carbon has more pores due to the advantages of natural porous structure of hemp stem. The aperture size is mainly microporous, and there are mesopores and macropores in the porous carbon. The porous carbon has an excellent reversible capacity of 495 mAh/g after 100 cycles at 0.2 °C as the anode of lithium-ion battery. Compared with the graphite electrode, the electrochemical property of activated carbon is significantly improved due to the reasonable distribution of pore size. The preparation of the activated carbon provides a new idea for low cost and rapid preparation of anode materials for high capacity lithium-ion batteries.

## Introduction

Although biomass wastes are high-value functional materials, a large amount of renewable agricultural wastes are limited exploited. It has been reported that biomass wastes are prepared as activated carbon and utilized as adsorbent material [1–4]. Vinod Kumar Gupta et al. [1] prepared activated carbon derived from *Ficus carica* fiber and applied it as a potential adsorbent for Cr (VI) removal, and the maximum adsorption capacity of Cr (VI) was 44.84 mg/g. Biomass wastes can also be used as hydrogen storage material [5–7]. W. Zhao et al. [5] prepared activated carbon with super surfaces areas of 3155 m^2^/g from bamboo doped with nitrogen. Absolutely, biomass carbon can also be used in supercapacitors [8, 9]. Youning Gong, Chunxu Pan et al. [8] synthesized three-dimensional porous graphitic biomass carbon and studied its electrochemical performance as electrode materials for supercapacitors. The electrode exhibited a high specific capacitance of 222 F/g at 0.5 A/g and studied its electrochemical performance as electrode materials for supercapacitors. It is worth mentioning that the anode material of lithium-ion batteries is an important application on functional materials [10–17]. Ran-Ran Yao et al. [10] synthesized hollow graphene sphere by oil bag emulsion liquid technology, which has good electrochemical properties of lithium storage. The high rate performance of hollow graphene spheres is due to the hollow structure, thin shells, and porous shells composed of graphene slices. Yi Li, Chun Li et al. [11] prepared a novel mesoporous activated carbon derived from corn stalk core by carbonization and KOH activation, which the BET surface area is 393.87 m^2^/g and the activated carbon anode possesses an excellent reversible capacity of 504 mAh/g after 100 cycles at 0.2 °C. In recent years, more and more achievements have been reported in the preparation of composite materials for carbon materials and the application of lithium-ion batteries [18–22]. Qigang Han, Zheng Yi et al. [18] prepared one-dimensional bioinspired bamboo carbon fiber and its composite. The composite is used as the anode of lithium-ion batteries, a high reversible capacity of 627.1 mAh/g is maintained over 100 cycles at a current density of 100 mAh/g. In general, the biomass wastes are promising for the preparation of energy-related materials, and it is of great significance to develop new waste resources legitimately.

Hemp is a green, sustainable, high-yield crop, and its sources will continue to expand in the background of the ever-opening of hemp cultivation. Nowadays, hemp is widely used in many fields. Thomas M. Attard et al. [23] obtained polymer CBD with high clinical therapeutic efficacy by Soxhlet extraction of hemp dust residue. Hemp can also be used as an aggregate for concrete [24, 25]. M. Rahim et al. [24] investigated thermal properties of three bio-based materials including hemp concrete, and the results showed that these building materials have an interesting heat storage capacity and a low thermal conductivity. Hom Nath Dhakal et al. [26] prepared biocomposites with poly (ε-caprolactone) and lignocellulosic hemp fiber by a twin extrusion process for lightweight applications. Besides, industrial cannabis can also be a precursor to ethanol production [27]. However, a limited hemp stem is rationally utilized under the condition of large-scale hemp cultivation. The industrial application of biomass waste hemp straw can not only reduce environmental pollution and resource waste caused by improper treatment of agricultural waste but also increases the added value of the corresponding industries. In addition, the application of hemp stems to lithium-ion batteries is a subject worth exploring.

In the previous reports, hemp stems exhibit splendid performance due to the natural porous property and excellent structure of hemp stems [28, 29]. Ru Yang, Jianchun Zhang et al. [30–32] prepared hemp stems derived activated carbon with high specific surface area by different activation method for adsorption materials and energy-related applications. MinHo Yang et al. [22] obtained 3D heterogeneous catalysts derived from vertical MnO_2_ wires deposited on hemp-derived 3D porous carbon by a one-step hydrothermal method. Wei Sun, Stephen M. Lipk et al. [33] prepared activated carbons derived from raw hemp stem (hurd and bast) via hydrothermal processing and chemical activation, and proposed a simple relationship between the specific area capacitance and the fraction of micropores by the rule of mixtures. Ji Zhang, Jianmin Gao et al. [34] prepared high surface area hemp stem-based activated carbon by KOH activation and investigated the influence of impregnation ratio, activation temperature, and activation time on AC specific surface area and reaction mechanism during material preparation. Shan Liu, Lei Ge et al. [35] prepared biomass carbon materials from hemp hurd and retted hemp hurd activated by CO_2_ or ZnCl_2_, which correspond to physical activation and chemical activation processes, respectively.

As a natural biomass resource, hemp stems are normally used for preparing porous carbon as adsorbent or hydrogen storage material [31, 35]. However, hemp stems are barely prepared as biomass porous carbon for lithium-ion batteries anode materials until now. In this paper, the advantage of hemp stems as lithium batteries anode materials are studied, which is induced by the porosity of hemp. Meanwhile, a new type of amorphous carbon is synthesized by pyrolysis and carbonization of hemp stems. The prepared ACs derived from hemp stems has an excellent electrochemical performance for anode of lithium-ion batteries. Due to its abundant resources and low preparation cost, we believe that it will be one of the promising electrode materials for lithium-ion batteries.

## Methods

### Preparation of Hemp Stems-Derived Activated Carbon

Raw hemp stems were obtained from the field of Heilongjiang Province. The peeled hemp stems were washed with deionized water, dried at 60 °C, and pulverized. A certain amount of powder was heated to 300 °C for 3 h under argon (inert gas) atmosphere at a rate of 5 °C/min for carbonization, while much tar is decomposed and released. The precursor was thoroughly mixed with ZnCl_2_ at the mass ratio of 1:5, and the mixture was placed in a tube furnace. The temperature was raised to 500–800 °C for 3 h and cooled to room temperature. After the activation product is ground, it is immersed in a 2-mol/L hydrochloric acid solution for 24 h to dissolve residual inorganic impurities, and then repeatedly washed with deionized water until the pH of the solution is 7 and dried. Hemp stems-derived activated carbon samples were denoted as AC-λ, where λ represented the activation temperature. The samples were subjected to a carbonization process and further processed at 600 °C without addition of ZnCl_2_, which were set as reference samples denoted as UAC.

### Materials Characterization

The powder X-ray diffraction (XRD) patterns were obtained on a Siemens D5000 X-ray Diffractometer with nickel-filtered Cu *Kα*1 radiation. Raman spectra were recorded on a Renishaw invia instrument. The morphology of the porous carbon was observed by scanning electron microscopy with field-emission-scanning electron microscope (JEOL JSM-6700F). The microstructure of the materials was examined by transmissions electron microscopy (JEM-2100F). The specific surface area and pore size distribution of the carbons were measured by nitrogen adsorption-desorption measurements (Micromeritics, ASAP2420).

### Electrochemical Measurements

The porous carbon, acetylene black, and polyvinylidene fluoride (PVDF) were evenly ground in a mortar at the mass ratio of 8:1:1 with an appropriate amount of *N*-methyl-2-pyrrolidone (NMP). The mixture was magnetically stirred for several hours to form a uniform slurry. The slurry was uniformly coated on a copper foil and dried in a vacuum oven at 120 °C for 12 h. The circular anode with a diameter of 10 mm was obtained by a tableting machine. The coin-type battery (CR2025) is assembled in an argon-filled glove box with a moisture and oxygen concentration of less than 0.1 ppm inside the cabinet. The lithium sheet is used as a counter electrode and a reference electrode, and the separator is polypropylene. The solvent in the electrolyte is a mixture containing EC, DMC, and EMC with a volume ratio of 1:1:1 dissolved in 1 M LiPF_6_. After assembly, the cycle performance test is performed by the LAND battery test system at a test voltage range of 0.02~3 V. The cyclic voltammetry (CV) curve and impedance test are performed on the electrochemical workstation.

## Results and Discussion

**Fig. 1:**
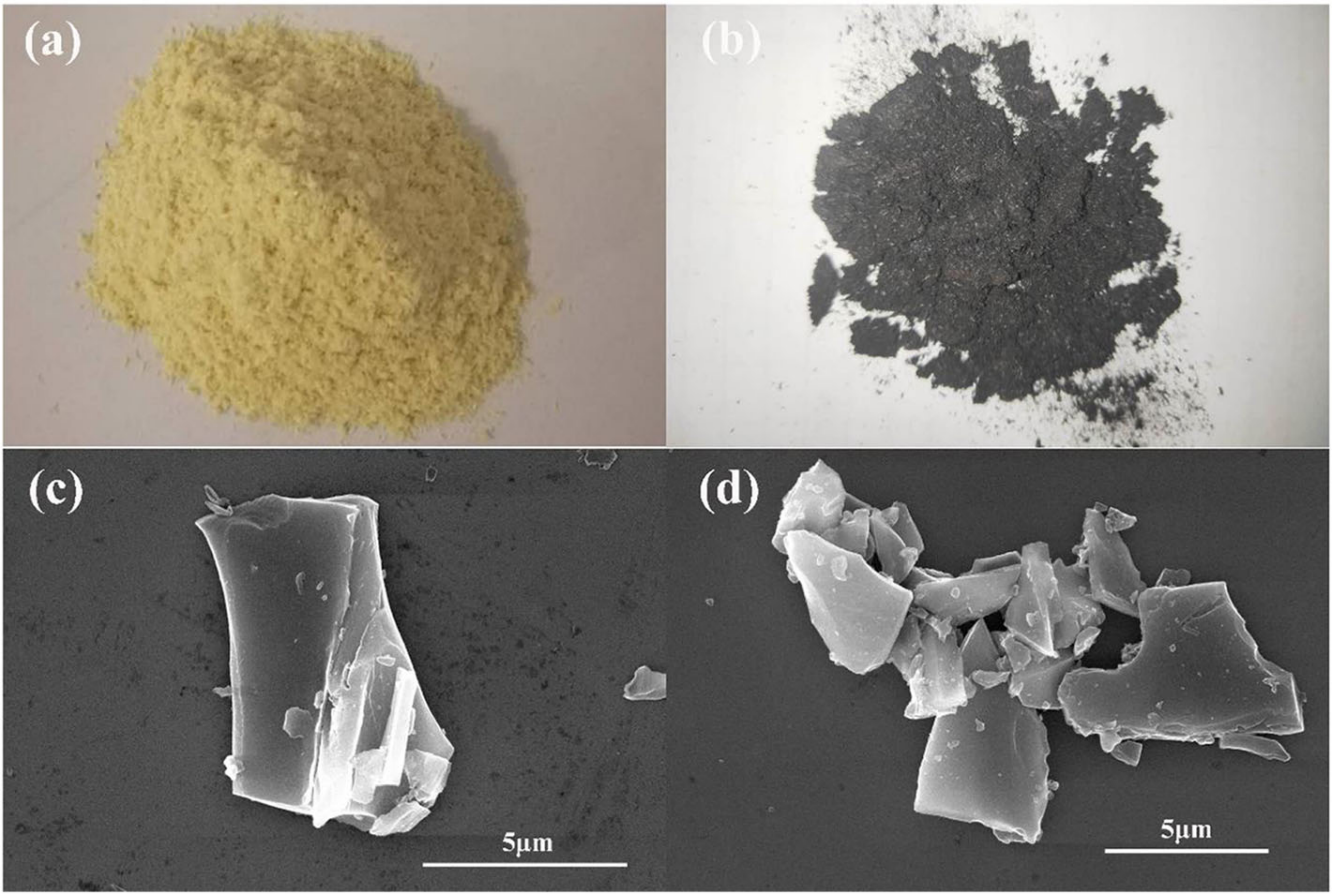
**a** Hemp stem powder. **b** Carbide of hemp stem. **c** SEM image of UAC. **d** SEM image of AC

**Fig. 2:**
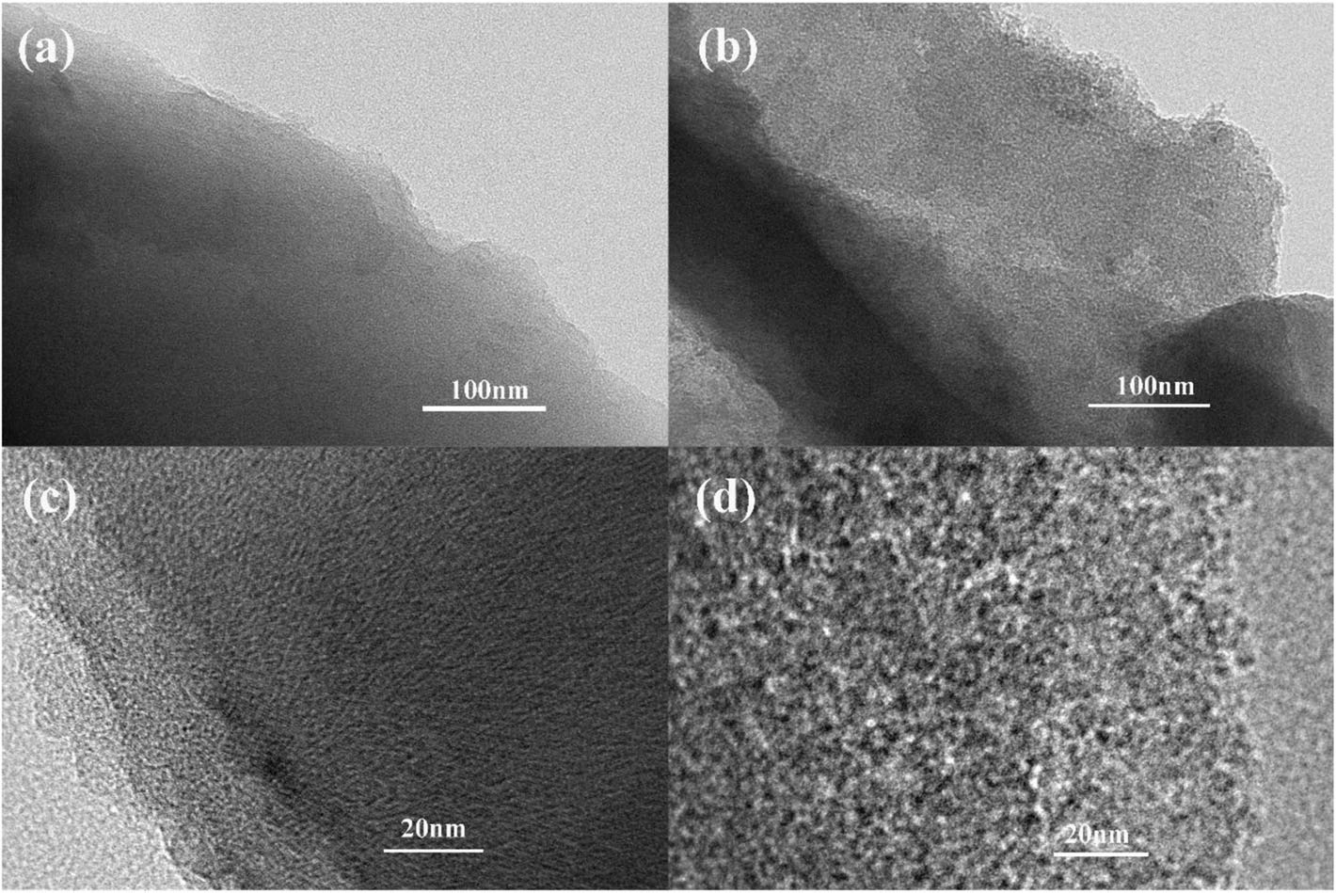
**a** TEM pattern of UAC. **b** TEM pattern of AC. **c** HRTEM pattern of UAC. **d** HRTEM pattern of AC

The hemp stems are pretreated to obtain the hemp stems powder as shown in Fig. 1a, and then carbonized to obtain the carbide as shown in Fig. 1b. As shown in Fig. 1c, d, the morphology of the UAC and AC-600 sample was characterized by SEM. Both the samples are amorphous carbon overall, no obvious macropore is observed. The role of the activator ZnCl_2_ is to promote pore formation and dissolve tar and other by-products [28]. The image also indicates that AC-600 is a complex of a large number of sheet-like structures and slit-like interspace, which will provide more active sites. Figure 2a, b shows TEM patterns of UAC and AC-600. Compared with UAC, AC has more obvious pores than UAC, results in providing more active sites and thus increasing the specific capacity of the batteries. Figure 2c, d depicts high-resolution TEM spectra of UAC and AC-600. It can be seen that UAC has pores at high magnification and is primarily microporous. Compared to UAC, AC-600 has more pores and larger pore sizes, indicating that the material has an excellent activation effect. In general, the porosity of AC is attributed to the natural internal porous structure of the hemp stems and the good activation effect of the activator.

**Fig. 3:**
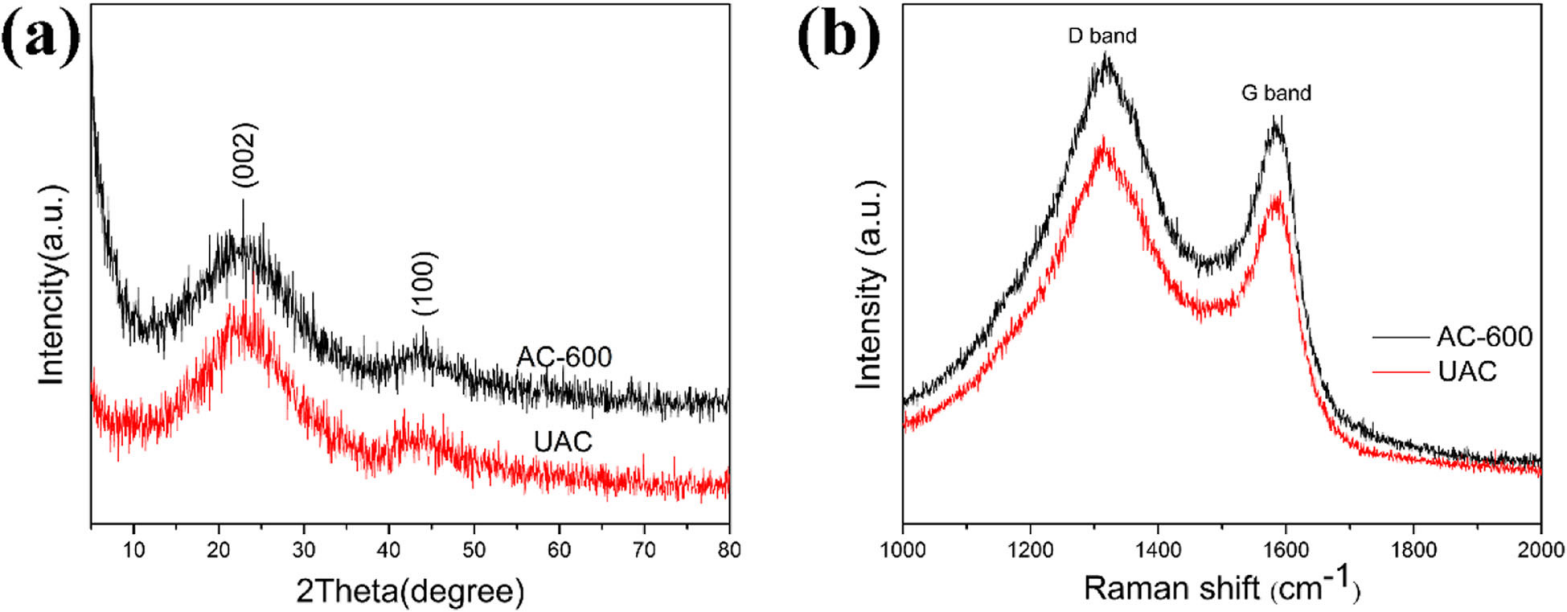
**a** X-ray diffraction patterns. **b** Raman spectrums of UAC and AC

The X-ray diffraction patterns of UAC and AC are shown in Fig. 3a. A broad diffraction peak around 22° corresponds to the (002) reflection of the graphite structure, which distributed to the presence of continuous parallel graphite sheets in the material. The relatively weak peak at 44° corresponding to the crystal plane (100) is considered honeycomb structures formed by sp2 hybridization [30, 31]. Besides, no sharp peaks were observed on these two diffraction peaks, indicating that both samples exhibit the out-of-order structure of disordered carbon material.

The Raman spectra of AC and UAC are shown in Fig. 3b. The D-band represents the disordered carbon layer structure and defects in the carbon material, and the G-band signifies the vibration of sp2 hybridized carbon atoms in the graphite sheet structure. Usually, *I*_D_/*I*_G_ is used to indicate the disorder degree of carbon. The *I*_D_/*I*_G_ of two carbon materials is 1.15 and 1.17, indicating that both have high amorphousness, more edges, and other defects. These features will provide more active sites for the insertion of lithium ions, which are of great benefit to improving the reversible capacity of the electrodes.

**Fig. 4:**
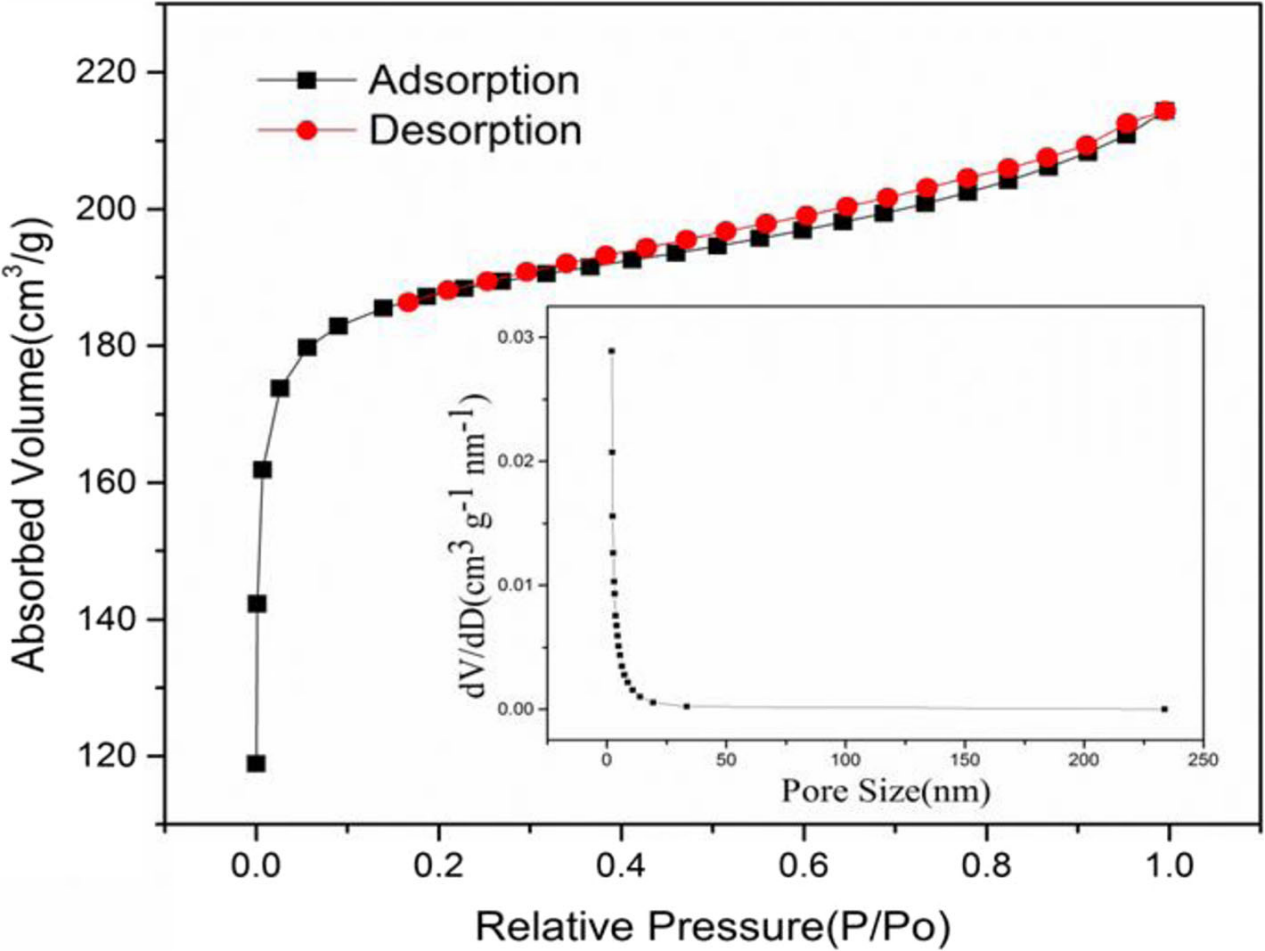
Isothermal adsorption-desorption curve of AC (illustration is pore size distribution)

The results of the surface area and pore size distribution of AC are shown in Fig. 4. The isotherm can be expressed as type I, indicating that the carbon material has plenty of micropores. The closed hysteresis loop of the adsorption-desorption isotherm can be classified as H4 type, indicating the presence of slit-like pores, which are formed by the accumulation of material debris particles. It delivers an excellent specific surface area that BET value is 589.54 m^2^/g. The pore size of AC is mainly distributed in the range of micropores that refers to pores of smaller than 2 nm, which is consistent with the results of the N_2_ adsorption-desorption isotherm. The pore volume and average pore diameter of AC were 0.332 cm^3^/g and 2.250 nm, respectively. There are not only many micropores, but also mesopores in the material, providing more active sites, and facilitating the cycling insertion and extraction of lithium ions. The transfer speed of ions is improved, and the impedance of batteries reduces [13].

In order to investigate the electrochemical behavior of the porous material, the material was analyzed by cycle stability performance, rate performance, impedance, and cyclic voltammetry (CV) tested for the anode of lithium-ion batteries.

**Fig. 5:**
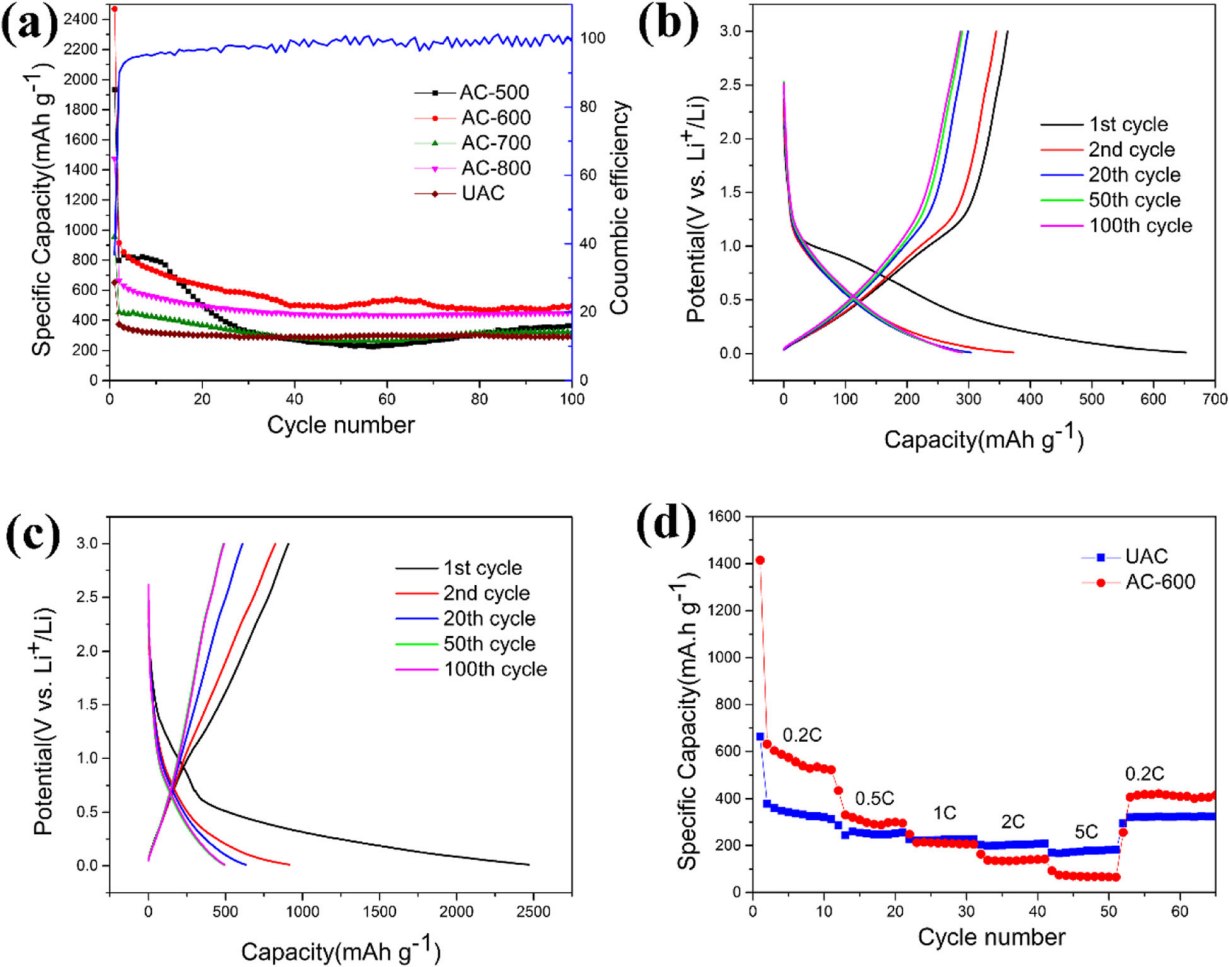
**a** Cycle performance curves of different materials. **b**, **c** Charge-discharge voltage curves of UAC and AC-600. **d** Rate performance of UAC and AC-600

Figure 5a shows that the charge-discharge cycle performance of activated carbon by different activation temperatures at a rate of 0.2 °C, in which the blue line corresponds to the Coulombic efficiency of AC-600. It delivers a distinguished capability clearly that the specific capacity of AC-600 is 495.4 mAh/g, which is much higher than the graphite theoretical capacity. The first discharge specific capacity and charge specific capacity are 2469.7 mAh/g and 1168.1 mAh/g, respectively. The first cycle has poor coulomb efficiency (only about 36%), which is consistent with the common characteristics of lithium-ion batteries cycle performance [15, 20]. The huge capacitance loss of the first cycle is attributed to the irreversible consumption of a large amount of lithium ions by the solid electrolyte interface (SEI) film forming on the electrode surface due to the large specific surface area. Its CE is around 100%, which denotes that AC-600 has a small capacity loss rate. The charge and discharge curves of the first cycle to the 100th cycle of UAC and AC-600 are shown in Fig. 5b, c. Both the charge capacity and the discharge capacity are gradually stabilized with the increase of the number of cycles. It can be found that the coincidence state of 50th and 100th charge-discharge profiles are perfectly impressive, indicating that the material has good stability in cycle performance.

The rate discharge performance of the as-prepared materials at current densities of 0.2 C–5 °C is shown in Fig. 5d. The AC-600 exhibits good rate ability with average discharge capacities of 522.6 mAh/g, 295.6 mAh/g, 205.4 mAh/g, 142.9 mAh/g, and 65.2 mAh/g at current densities of 0.2 °C, 0.5 °C, 1 °C, 2 °C, and 5 °C, separately. The initial performance of the AC-600 is higher, and the capacity drops significantly at larger magnifications, but when the discharge rate is restored to 0.2 °C, the performance of AC-600 can still be restored to a higher reversible capacity of 416.3 mAh/g. Conversely, the initial capacity of UAC is lower, but the capacity decreases less at large rates. The UAC exhibits average discharge capacities of 313.3 mAh/g, 255.7 mAh/g, 227.1 mAh/g, 209.2 mAh/g, 181.7 mAh/g, and 323.5 mAh/g at same current densities as AC-600. Although it has a lower specific capacity than AC-600, it exhibits good capacity retention. This phenomenon can be attributed to the large specific surface area of AC-600 caused by the activation process, so that the specific surface area in contact with lithium ions increases. As the electrochemical cycle progress proceeds, large side reactions consume a large amount of lithium ions and are irreversible, resulting in a decrease in capacity.

**Fig. 6:**
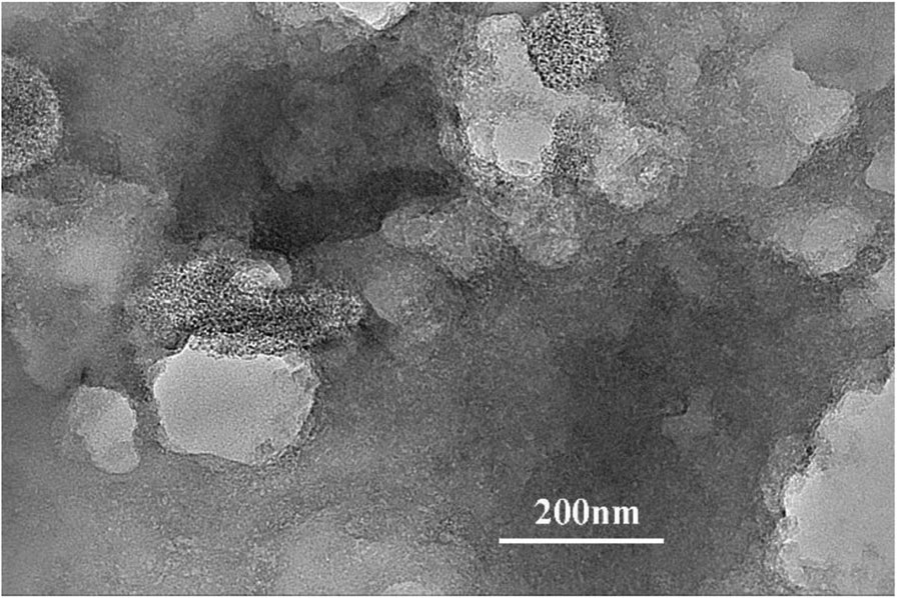
TEM pattern of spent electrode materials after cycling

In order to further confirm the origin of the good performance of AC-600 and also to identify the possible reasons for performance fading, the TEM spectrum of the spent electrode material after cycling was measured. As shown in Fig. 6, partial surface of the AC-600 is actually broken after cycling, exposing the internal porous structure. This may be attributed to the excessive activation effect that occurs on the surface of the carbon material. Partial surface damage and SEI re-formation occur during cyclic insertion-extraction of lithium ions.

**Fig. 7:**
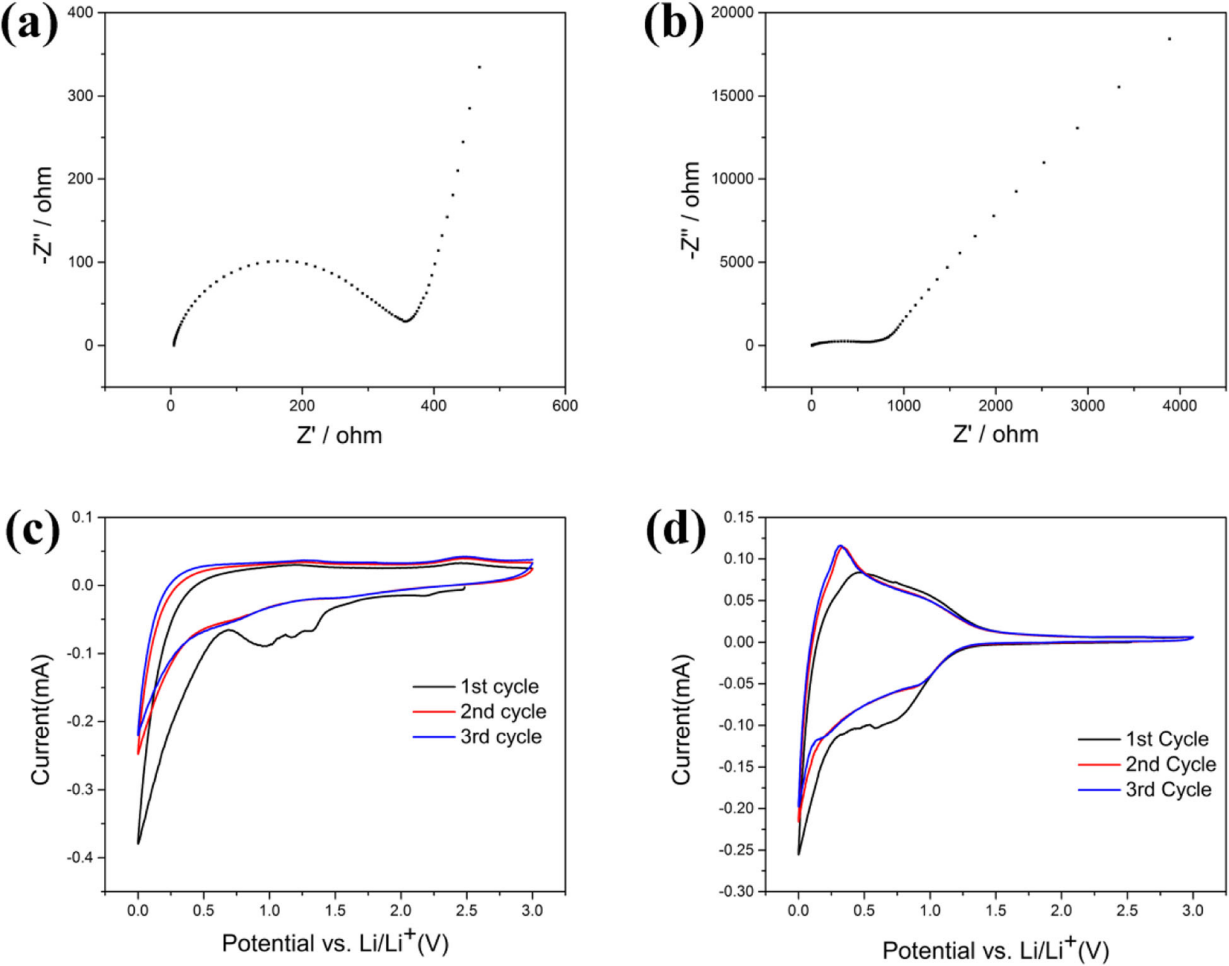
**a** Impedance spectrums of AC-600. **b** Impedance spectrums of UAC. **c** Cyclic voltammogram profiles of AC-600. **d** Cyclic voltammogram profiles of UAC

The impedance spectrum of the samples was tested to reveal the kinetics of the electrodes during ion transport, as shown in Fig. 7a, b. The high-frequency semicircle corresponds to the contact resistance. The semicircle of the intermediate frequency region is attributed to the charge transfer impedance at the electrode/electrolyte interface. The oblique line at an angle of about 45° to the real axis corresponds to the lithium-ion diffusion process in the carbon electrode [32]. No obvious semicircle is observed in the impedance spectra of UAC due to the large resistance of UAC. Conversely, the impedance map of AC-600 exhibits a relatively obvious semicircle. This is attributed to the large pore distribution inside the activated sample, which promotes the transport of lithium ions and accelerates the timely embedding and escaping of ions in the anode material. The initial 3 cycles of cyclic voltampere (CV) curves at a scan rate of 0.1 mV/s between 0.01 and 3.0 V are displayed in Fig. 7c, d. In the reduction process of the first circle, there is a sharp peak around 0.7 V and a weak peak around 1.35 V. For two samples, the cathodic peak at 1.35 V indicated that an irreversible reaction has begun between electrode and electrolyte [18]. The peak around 0.7 V is due to the decomposition of the electrolyte on the electrode surface and the formation of the solid electrolyte interface (SEI) film. These peaks disappeared in the subsequent second and third cycles, indicating that the above reactions in the first cycle are irreversible. In the first cycle, the lithium deintercalation process occurs at anodic peak around 0.25 V, which is consistent with many reported carbon substances [8, 18]. The difference is that the lithium deintercalation process of UAC is faster at low corresponding voltages while the reaction of AC-600 is flatter at the whole process. In the case excluding that UAC is hardly a mesoporous or macroporous structure, it can be reasonably concluded that the surface pores of the UAC are more combined with lithium ions, resulting in faster lithium removal of UAC during charging. Both AC-600 and UAC have a tendency to gradually coincide with the subsequent second and third cycles, and the second and third circles are substantially completely coincident in the figure, indicating that the electrode material has good stability.

## Conclusions

In conclusion, hemp stems-based activated carbon is applied in the anode of lithium-ion batteries, which provides a new idea for the industrialization preparation of low-cost and high-capacity hemp stems-based anode materials. The hemp stems-derived biomass carbon material obtained by carbonization and activation is a typical amorphous carbon. The activated carbon has a relatively obvious pore structure its BET surface area reaches 589.54 m^2^/g, and the pore diameter mainly exists in the form of micropores. The activated carbon as anode material achieved a high reversible capacity of 495 mAh/g after 100 cycles at 0.2 °C. The electrochemical performance of activated carbon is significantly improved compared to unactivated carbon. Although the sample prepared by the activation method has inherent defects of much ash, the production of volatile substances such as tar and highly corrosive chemicals to equipment, it still provides a new path for the high-value-added development and comprehensive utilization of biomass waste hemp stems. This method provides an effective method for the rapid and low-cost preparation of anode materials and the comprehensive utilization of hemp stems.

## Data Availability

The conclusions made in this manuscript are based on the data which are all presented and shown in this paper.

## Abbreviations

AC: Activated carbon
CE: Coulomb efficiency
CV: Cyclic voltammetry
DMC: Dimethyl carbonate
EC: Ethylene carbonate
EMC: Ethyl methyl carbonate
SEI: Solid electrolyte interface
UAC: Unactivated carbon
